# Blastomycosis, Histoplasmosis, and Coccidioidomycosis in Outpatient Community-Acquired Pneumonia

**DOI:** 10.1001/jamanetworkopen.2025.53965

**Published:** 2026-01-14

**Authors:** Kaitlin Benedict, George R. Thompson, Neil M. Ampel, Dallas J. Smith, Mitsuru Toda, Ian Hennessee

**Affiliations:** 1 Mycotic Diseases Branch, Division of Foodborne, Waterborne, and Environmental Diseases, National Center for Emerging and Zoonotic Infectious Diseases, Centers for Disease Control and Prevention, Atlanta, Georgia; 2 Department of Medicine, Division of Infectious Diseases, University of California, Davis, Sacramento; 3 Department of Medical Microbiology and Immunology, University of California, Davis, Sacramento; 4 Department of Medicine, University of Arizona, Tucson; 5 Department of Immunobiology, University of Arizona, Tucson

## Abstract

**Importance:**

Laboratory testing is necessary to distinguish blastomycosis, coccidioidomycosis, or histoplasmosis from other causes of community-acquired pneumonia (CAP). Robust data about testing for and diagnosis of these fungal diseases among patients with CAP throughout the US are lacking.

**Objective:**

To examine proportions and characteristics of (1) adult outpatients with CAP who underwent diagnostic testing for blastomycosis, coccidioidomycosis, or histoplasmosis; and (2) tested patients who received diagnoses of these diseases.

**Design, Setting, and Participants:**

This retrospective cohort study used 2017 to 2023 commercial health insurance claims data from the Merative MarketScan Commercial/Medicare Database. Adult outpatients with diagnosis codes for unspecified CAP were included.

**Main Outcomes and Measures:**

The primary outcome was the proportion of patients who underwent fungal diagnostic testing. The secondary outcomes were the proportions of tested patients who received diagnoses of blastomycosis, coccidioidomycosis, or histoplasmosis. Multivariable logistic regression models were used to estimate adjusted odds ratios (aORs) for characteristics independently associated with receiving a diagnosis of coccidioidomycosis or histoplasmosis.

**Results:**

Among 573 994 patients (318 152 female [55%]; median [IQR] age, 54 [41-63] years) with unspecified CAP, 25 822 (5%) underwent fungal diagnostic testing, which occurred a median (IQR) of 3 (1-6) health care visits after the initial CAP diagnosis. Among tested patients, 755 (3%) received a blastomycosis, coccidioidomycosis, or histoplasmosis diagnosis code. Rash (aOR, 3.24; 95% CI, 2.27-4.63), lymphadenopathy (aOR, 1.74; 95% CI, 1.15-2.63), myalgia (aOR, 1.66; 95% CI, 1.11-2.49), chest pain (aOR, 1.65; 95% CI, 1.35-2.02), and receipt of antibiotics from multiple classes (aOR, 1.40; 95% CI, 1.17-1.67) were independently associated with increased odds of receiving a coccidioidomycosis diagnosis. Autoimmune inflammatory disease (aOR, 3.00; 95% CI, 1.72-5.21), chest pain (aOR, 1.84; 95% CI, 1.20-2.82), and abnormal weight loss (aOR, 5.15; 95% CI, 2.71-9.79) were associated with increased odds of receiving a histoplasmosis diagnosis.

**Conclusions and Relevance:**

In this cohort study of patients with unspecified CAP, testing rates for blastomycosis, coccidioidomycosis, and histoplasmosis were low in many locations. Increased awareness of these fungal infections may help increase timely testing for fungal diseases among patients with CAP, decrease health care utilization and inappropriate antibiotic use, and improve patient outcomes.

## Key Points

**Question:**

What proportion of adult outpatients with unspecified community-acquired pneumonia (CAP) underwent diagnostic testing for blastomycosis, coccidioidomycosis, or histoplasmosis, and what proportion tested positive for those diseases?

**Findings:**

In this cohort study of health insurance claims data, among 573 994 patients with unspecified CAP, 5% underwent fungal diagnostic testing, after a median of 3 health care visits. Among tested patients, 3% received a blastomycosis, coccidioidomycosis, or histoplasmosis diagnosis code.

**Meaning:**

These low testing rates highlight the potential for missed diagnoses of blastomycosis, coccidioidomycosis, and histoplasmosis; increased testing could lead to decreased health care utilization and inappropriate antibiotic use, and improve patient outcomes.

## Teaser

This cohort study examines the characteristics of adult outpatients with community-acquired pneumonia who underwent diagnostic testing for blastomycosis, coccidioidomycosis, or histoplasmosis and received diagnoses for these diseases.

## Introduction

Blastomycosis, coccidioidomycosis, and histoplasmosis are diseases primarily acquired via inhalation of environmental fungal pathogens in certain geographic regions of the US. Infection can range from asymptomatic to life-threatening disseminated disease; however, community-acquired pneumonia (CAP) is a common presentation for each of these fungal diseases. Given their clinical similarity to bacterial or viral CAP, laboratory testing is necessary to distinguish blastomycosis, coccidioidomycosis, and histoplasmosis from other CAP causes.^1^

National guidelines for CAP diagnosis and treatment from the American Thoracic Society and Infectious Diseases Society of America recommend initial empiric antibiotic treatment for bacterial infection among adults with CAP who do not have an immunocompromising condition.^2^ Diagnostic testing to determine the cause of CAP is recommended in specific clinical situations; however, the current guidelines do not describe indications for testing for fungal diagnostic testing, despite recommending testing for other relatively uncommon causes of CAP, such as *Legionella* species, when implicated by epidemiologic evidence or in severe CAP cases.^2^ Separate clinical diagnostic guidance for blastomycosis, coccidioidomycosis, and histoplasmosis suggests testing for these diseases in patients with CAP with exposure to known endemic areas and symptoms that did not improve following empiric antibiotics.^1^ However, primary care practitioners self-report infrequently testing for these diseases, even in areas where they are most common.^3^ Consequently, patients with blastomycosis, coccidioidomycosis, and histoplasmosis often receive a diagnosis from specialist health care practitioners after multiple antibiotic courses and substantial diagnostic delays, often exceeding 1 month.^4,5,6,7,8^ These delays result in unnecessary antibiotic use, increased health care costs, and potentially worse patient outcomes.^4,5,9^

Robust data about fungi as a cause of CAP in the US are lacking.^10^ Data on proportions and characteristics of patients with CAP tested for and receiving a diagnosis of fungal diseases could help inform the true proportion of CAP due to fungi. Studies designed to overcome the clinical practice limitations of undertesting by actively testing patients show that these fungal diseases are common in certain areas. Two studies found that coccidioidomycosis caused 17% and 29% of CAP in Phoenix and Tucson, Arizona, respectively, and another showed that among patients hospitalized with acute respiratory infections in Houston, Texas, 13% had a positive *Coccidioides* antibody test and 8% had a positive *Histoplasma* immunodiagnostic test; however, those studies were based on small sample sizes and were geographically limited.^11,12,13^ We used a large commercial health insurance claims database to examine proportions and characteristics of (1) patients with unspecified CAP who underwent fungal-specific diagnostic testing, and (2) among those tested, patients who received diagnoses of blastomycosis, coccidioidomycosis, or histoplasmosis. A secondary objective was to compare frequencies of blastomycosis, coccidioidomycosis, and histoplasmosis with other less-common CAP causes for which American Thoracic Society guidelines recommend diagnostic testing in specific clinical situations.

## Methods

The Merative MarketScan Commercial/Medicare Database^14^ contains health insurance claims data, including inpatient and outpatient visits and outpatient prescriptions for more than 54 million employees, dependents, and retirees with employer-sponsored plans, including Medicare Supplemental and Medicare Advantage plans throughout the US during 2017 to 2023. MarketScan data are fully deidentified, so this cohort study was not subject to review by the Centers for Disease Control and Prevention institutional review board (see 45 CFR part 46, 21 CFR part 56; 42 USC §241(d); 5 USC §552a; and 44 USC §3501 et seq). This study was conducted in accordance with the Strengthening the Reporting of Observational Studies in Epidemiology (STROBE) reporting guidelines for cohort studies.

We identified adults aged 18 years and older who had an *International Statistical Classification of Diseases, Tenth Revision, Clinical Modification (ICD-10-CM)* code for unspecified CAP (eTable in Supplement 1) at an outpatient visit during January 1, 2017, to December 31, 2023 (study window). The index date was the date the CAP diagnosis code was first used during the study window. We selected patients with continuous insurance enrollment during the 90 days before and 90 days after the index date and limited the cohort to patients who received a prescription for systemic outpatient antibiotics during the 14 days before and 14 days after the index date.^6^ Patients who were hospitalized in the 14 days before the index date were excluded to minimize capturing hospital-associated pneumonia. Patients who received diagnoses of blastomycosis, coccidioidomycosis, or histoplasmosis in the 90 days before the index date were also excluded, to focus on incident diagnoses of these diseases.

We examined demographic characteristics and select underlying conditions, signs, symptoms, health care practitioner type, and diagnostic testing for blastomycosis, coccidioidomycosis, or histoplasmosis. Diagnostic testing was identified using *Current Procedural Terminology (CPT)* codes and included fungal culture, microscopy, and serology within 90 days after the index date. We examined proportions of patients who received an *ICD-10-CM* diagnosis code for blastomycosis, coccidioidomycosis, or histoplasmosis within 90 days after the index date, excluding rule-out diagnoses listed on a laboratory or imaging claim only. For comparison, we similarly examined proportions of patients who received an *ICD-10-CM* diagnosis code for legionellosis, pneumococcal pneumonia, *Pseudomonas aeruginosa* pneumonia, and methicillin-resistant *Staphylococcus aureus* (MRSA) pneumonia in the 90 days after the index date. These proportions were examined independently of whether patients received a *CPT* code for compatible diagnostic testing given the lack of specificity within the *CPT* coding scheme.

### Statistical Analysis

We calculated overall and state-specific (based on the insurance policy holder’s residence state) proportions of patients with unspecified CAP who underwent fungal diagnostic testing and who received a blastomycosis, coccidioidomycosis, or histoplasmosis diagnosis code. We conducted bivariate analyses to compare demographic and clinical features among patients who did vs did not undergo fungal diagnostic testing and among tested patients who did vs did not receive a blastomycosis, coccidioidomycosis, or histoplasmosis diagnosis code, using χ^2^ or Fisher exact tests for categorial variables and Wilcoxon rank-sum tests for continuous variables. The primary outcomes for these analyses were binary indicators of (1) receipt of a fungal-specific diagnostic test and (2) receipt of a diagnosis code for blastomycosis, coccidioidomycosis, or histoplasmosis. Bivariate comparisons significant at *P* < .20 were considered for inclusion in 3 separate backward multivariable logistic regression models to estimate adjusted odds ratios (aORs) and 95% CIs for characteristics independently associated (ie, adjusted for covariates) with the following outcomes: (1) undergoing a fungal-specific test, (2) receiving a diagnosis of coccidioidomycosis, and (3) receiving a diagnosis of histoplasmosis. We were unable to assess characteristics associated with blastomycosis diagnosis because of small sample size. All analyses were conducted using the MarketScan Treatment Pathways online tool and SAS statistical software version 9.4 (SAS Institute). Two-sided *P* < .05 was considered statistically significant.

## Results

### CAP Cohort

Among 1 143 261 adult outpatients with unspecified CAP, 840 948 met continuous enrollment criteria; 626 944 received antibiotics; 347 were excluded because of a previous blastomycosis, coccidioidomycosis, or histoplasmosis diagnosis; and 52 603 were excluded because of a previous hospitalization, leaving 573 994 in the final CAP cohort. The median (IQR) age of patients with unspecified CAP was 54 (41-63) years; 318 152 (55%) were female, and 246 364 (43%) visited a family practitioner or internal medicine practitioner on the index date. Census region data were available for 539 793 patients, and 236 844 (44%) were from the South Census region. In total, 181 772 patients (32%) with unspecified CAP received antibiotics from multiple classes (Table 1).

**Table 1.** Characteristics of Outpatients With Unspecified CAP, by Undergoing Any Diagnostic Test for Blastomycosis, Coccidioidomycosis, or Histoplasmosis

Characteristic	Patients with unspecified CAP, No. (%)			aOR (95% CI)^a^
	Total (N = 573 994)	Tested (n = 25 822)	Not tested (n = 548 172)	
Demographic characteristics				
Age, median (IQR), y	54 (41-63)	55 (43-63)	54 (41-63)	NA
Age group, y				
18-44	174 047 (30)	7086 (27)	166 961 (30)	1 [Reference]
45-64	275 229 (48)	13 571 (53)	261 658 (48)	1.03 (1.00-1.07)
≥65	124 718 (22)	5165 (20)	119 553 (22)	0.68 (0.64-0.71)
Sex				
Male	255 842 (45)	11 463 (44)	244 379 (45)	NA
Female	318 152 (55)	14 359 (56)	303 793 (55)	NA
Census region (n = 539 793)				
Midwest	146 686 (27)	6776 (27)	139 910 (27)	1.05 (1.01-1.09)
Northeast	82 522 (15)	3035 (12)	79 487 (15)	0.92 (0.88-0.96)
South	236 844 (44)	10 287 (42)	226 557 (44)	1 [Reference]
West	73 741 (14)	4649 (19)	69 092 (13)	1.62 (1.56-1.68)
Urban vs rural classification (n = 434 778)				
Nonrural	353 259 (81)	17 096 (83)	336 163 (81)	1 [Reference]
Rural	81 519 (19)	3544 (17)	77 975 (19)	0.86 (0.82-0.89)
Index date season				
Spring	143 567 (25)	6548 (25)	137 019 (25)	1 [Reference]
Summer	116 141 (20)	6186 (24)	109 955 (20)	1.11 (1.06-1.16)
Fall	139 587 (24)	6514 (25)	133 073 (24)	1.06 (1.01-1.10)
Winter	174 699 (30)	6574 (25)	168 125 (31)	0.85 (0.82-0.88)
Underlying conditions on or in the 90 d before index date				
Asthma	59 134 (10)	3836 (15)	55 298 (10)	1.24 (1.18-1.29)
Autoimmune inflammatory disease	22 652 (4)	1642 (6)	21 010 (4)	1.27 (1.20-1.36)
Chronic obstructive pulmonary disease or other chronic lower respiratory disease	76 180 (13)	5424 (21)	70 756 (13)	1.36 (1.31-1.42)
Diabetes	84 343 (15)	4630 (18)	79 713 (15)	1.07 (1.03-1.11)
Hyperlipidemia	77 392 (13)	4557 (18)	72 835 (13)	1.07 (1.03-1.12)
Hypertension	175 783 (31)	9485 (37)	166 298 (30)	NA
Hypothyroidism	41 158 (7)	2441 (9)	38 717 (7)	1.14 (1.08-1.20)
Immunosuppression	52 053 (9)	4440 (17)	47 613 (9)	1.51 (1.44-1.58)
HIV	2082 (<1)	203 (1)	1879 (<1)	NA
Cancer	32 359 (6)	2728 (11)	29 631 (5)	NA
Immunosuppressive medication besides prednisone^b^	9336 (2)	947 (4)	8389 (2)	NA
Prednisone (≥21 d supply)	11 980 (2)	1209 (5)	10 771 (2)	NA
Solid organ or stem cell transplant	4457 (1)	527 (2)	3930 (1)	NA
Overweight and obesity	56 895 (10)	3471 (13)	53 424 (10)	1.09 (1.04-1.14)
Pregnancy	2479 (<1)	170 (1)	2309 (<1)	1.45 (1.21-1.73)
Smoking (current or past)	66 603 (12)	4454 (17)	62 149 (11)	1.05 (1.01-1.10)
Selected symptoms and clinical findings on or in the 90 d before index date				
Symptoms				
Abnormal weight loss	5008 (1)	483 (2)	4525 (1)	1.49 (1.33-1.68)
Chest pain	95 894 (17)	6290 (24)	89 604 (16)	1.07 (1.03-1.10)
Chills, without fever	4549 (1)	273 (1)	4276 (1)	NA
Cough	252 508 (44)	12 444 (48)	240 064 (44)	NA
Dyspnea	138 572 (24)	9330 (36)	129 242 (24)	1.10 (1.06-1.13)
Erythema nodosum	81 (<1)	21 (<1)	60 (<1)	4.57 (2.53-8.26)
Fatigue or malaise	48 190 (8)	2894 (11)	45 296 (8)	1.12 (1.07-1.18)
Fever	93 593 (16)	5463 (21)	88 130 (16)	1.12 (1.08-1.16)
Generalized hyperhidrosis	2584 (<1)	228 (1)	2356 (<1)	1.53 (1.31-1.80)
Myalgia	18 072 (3)	943 (4)	17 129 (3)	NA
Pain in joint	56 681 (10)	3123 (12)	53 558 (10)	1.08 (1.03-1.13)
Rash	6696 (1)	600 (2)	6096 (1)	1.63 (1.47-1.80)
Clinical findings				
Acute respiratory failure	19 931 (3)	1861 (7)	18 070 (3)	1.14 (1.07-1.22)
Diseases of mediastinum	402 (<1)	66 (<1)	336 (<1)	1.50 (1.09-2.07)
Enlarged lymph nodes	8641 (2)	1103 (4)	7538 (1)	1.59 (1.47-1.72)
Eosinophilia	16 187 (3)	1670 (6)	14 517 (3)	1.47 (1.38-1.56)
Hypoxemia	22 840 (4)	1977 (8)	20 863 (4)	1.15 (1.08-1.23)
Other nonspecific abnormal finding of lung field	103 427 (18)	8293 (32)	95 134 (17)	1.34 (1.29-1.38)
Pleural effusion	25 762 (4)	2607 (10)	23 155 (4)	1.47 (1.39-1.55)
Solitary pulmonary nodule	11 440 (2)	1334 (5)	10 106 (2)	1.48 (1.38-1.60)
No. of symptoms and clinical findings, median (IQR)	1 (0-2)	2 (1-3)	1 (0-2)	NA
Practitioner type on the index date^c^				
Family practitioner or internal medicine	246 364 (43)	10 487 (41)	235 877 (43)	1.29 (1.24-1.33)
Pulmonologist	8978 (2)	1564 (6)	7414 (1)	3.94 (3.67-4.24)
Nurse practitioner or physician assistant	71 346 (12)	2532 (10)	68 814 (13)	NA
Acute care hospital	208 900 (36)	14 628 (57)	194 272 (35)	1.86 (1.79-1.92)
Infectious disease specialist	2166 (<1)	350 (1)	1816 (<1)	2.66 (2.31-3.05)
Emergency department or urgent care	169 556 (30)	8772 (34)	160 784 (29)	1.15 (1.11-1.20)
None of the above practitioner types	62 002 (11)	2234 (9)	59 828 (11)	1.45 (1.36-1.54)
Received antibiotics from >1 class^d^	181 772 (32)	11 259 (44)	170 513 (31)	1.56 (1.51-1.60)
Underwent chest imaging	367 419 (64)	20 224 (78)	347 195 (63)	1.54 (1.48-1.60)

Abbreviations: aOR, adjusted odds ratio; CAP, community-acquired pneumonia; NA, not applicable.

^a^ Unadjusted *P* < .001 for all comparisons except sex (*P* = .55).

^b^ Includes tumor necrosis factor–α inhibitors, interleukin-17 and interleukin-23 inhibitors, mycophenolate mofetil, and tacrolimus.

^c^ Categories are not mutually exclusive.

^d^ Includes macrolides (275 230 patients [48%]), quinolones (149 321 patients [26%]), tetracyclines (142 212 patients [25%]), penicillin (115 342 patients [20%]), cephalosporins (81 061 patients [14%]), sulfonamides (10 593 patients [2%]), and other (7221 patients [1%]).

### Fungal Diagnostic Testing

Overall, 25 822 patients (5%) with unspecified CAP underwent fungal diagnostic testing. By state, the highest proportions of patients with unspecified CAP who underwent fungal testing were in Arizona (1497 patients [17%]), North Dakota (21 patients [6%]), California (1805 patients [6%]), Florida (1961 patients [6%]), and Illinois (829 patients [5%]) (Figure). Fungal testing occurred a median (IQR) of 8 (1-37) days and a median (IQR) of 3 (1-6) health care visits after the index date.

**Figure.**
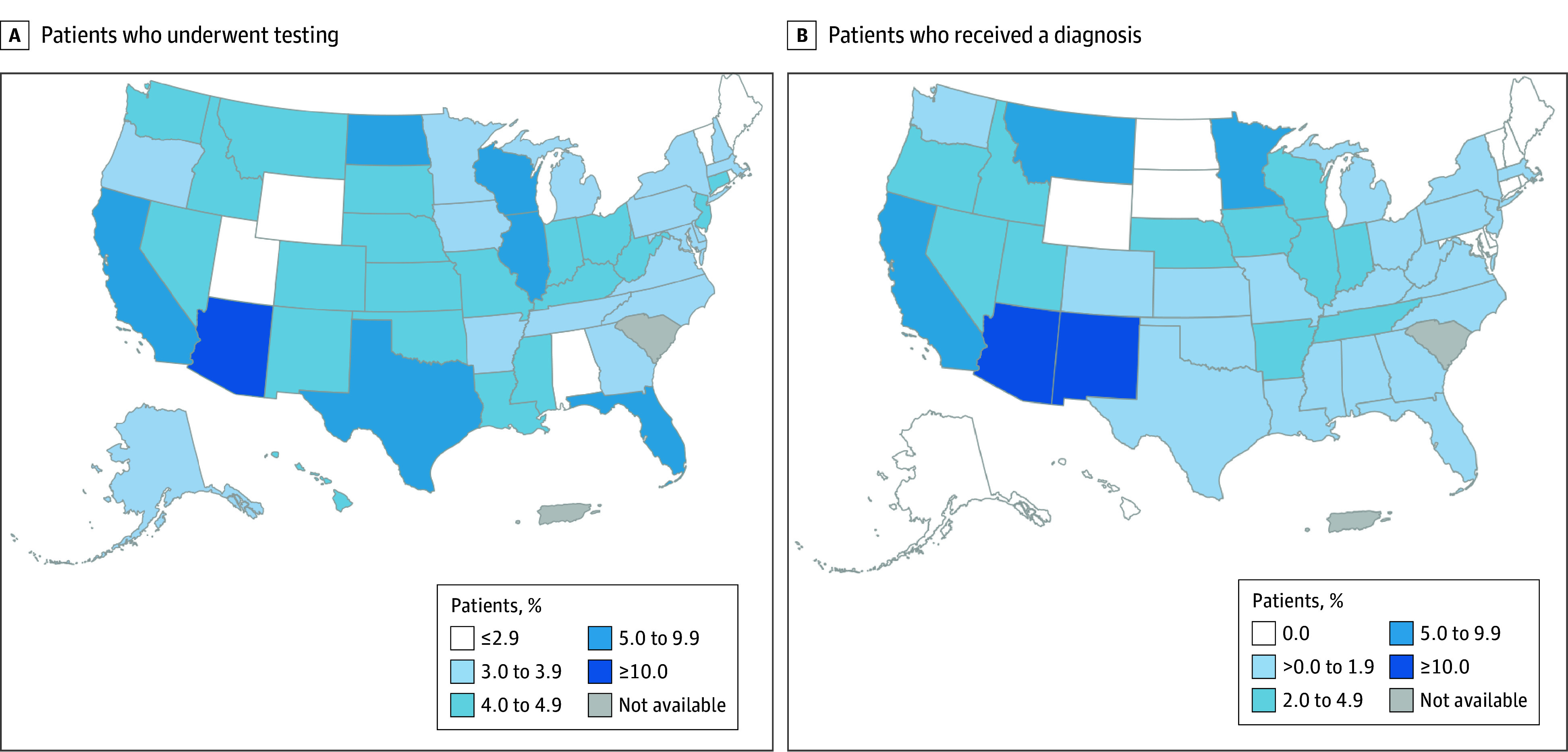
Outpatients With Unspecified Community-Acquired Pneumonia Maps show proportions of patients who underwent a diagnostic test for blastomycosis, coccidioidomycosis, or histoplasmosis (A), and, among tested patients, the proportion who received a diagnosis of blastomycosis, coccidioidomycosis, or histoplasmosis (B).

On multivariable analysis, the odds of undergoing fungal testing were lower for patients aged 65 years or older (aOR, 0.68; 95% CI, 0.64-0.71; reference group, 18-44 years) and for patients in the West (aOR, 1.62; 95% CI, 1.56-1.68; reference group, South) and the Midwest (aOR, 1.05; 95% CI, 1.01-1.09). All of the underlying conditions we examined were associated with higher odds of undergoing fungal testing, particularly immunosuppression (aOR, 1.51; 95% CI, 1.44-1.58), chronic obstructive pulmonary disease (COPD) (aOR, 1.36; 95% CI, 1.31-1.42), and autoimmune inflammatory disease (aOR, 1.27; 95% CI, 1.20-1.36) (Table 1).

Symptoms and clinical findings associated with highest odds of receiving fungal testing included erythema nodosum (aOR, 4.57; 95% CI, 2.53-8.26), rash (aOR, 1.63; 95% CI, 1.47-1.80), lymphadenopathy (aOR, 1.59; 95% CI, 1.47-1.72), generalized hyperhidrosis (aOR, 1.53; 95% CI, 1.31-1.80), abnormal weight loss (aOR, 1.49; 95% CI, 1.33-1.68), and presence of a solitary pulmonary nodule (aOR, 1.48; 95% CI, 1.38-1.60). Patients who sought care at a pulmonologist (aOR, 3.94; 95% CI, 3.67-4.24) or an infectious disease specialist (aOR, 2.66; 95% CI, 2.31-3.05) on the index date and received antibiotics from multiple classes (aOR, 1.56; 95% CI, 1.51-1.60) also had increased odds of undergoing fungal testing (Table 1).

### Diagnosis of Blastomycosis, Coccidioidomycosis, or Histoplasmosis

Among patients who underwent fungal testing, 755 (3%) received a diagnosis code for blastomycosis (51 patients), histoplasmosis (94 patients), or coccidioidomycosis (616 patients). States with the highest proportion of patients who received diagnoses were Arizona (344 patients [23%]), New Mexico (4 patients [11%]), California (174 patients [10%]), Montana (1 patient [5%]), and Minnesota (12 patients [5%]) (Figure). The median (IQR) time from index date to diagnosis was 20 (9-37) days.

On multivariable analysis, COPD (aOR, 0.42; 95% CI, 0.29-0.61) immunosuppression (aOR, 0.49; 95% CI, 0.35-0.69), and diabetes (aOR, 0.72; 95% CI, 0.57-0.91) were associated with reduced odds of receiving a coccidioidomycosis diagnosis code (Table 2). Symptoms and clinical characteristics associated with increased odds of receiving a coccidioidomycosis diagnosis code included rash (aOR, 3.24; 95% CI, 2.27-4.63), lymphadenopathy (aOR, 1.74; 95% CI, 1.15-2.63), myalgia (aOR, 1.66; 95% CI, 1.11-2.49), chest pain (aOR, 1.65; 95% CI, 1.35-2.02), and receipt of antibiotics from multiple classes (aOR, 1.40; 95% CI, 1.17-1.67). Patient characteristics associated with increased odds of receiving a histoplasmosis diagnosis code included autoimmune inflammatory disease (aOR, 3.00; 95% CI, 1.72-5.21), chest pain (aOR, 1.84; 95% CI, 1.20-2.82), and abnormal weight loss (aOR, 5.15; 95% CI, 2.71-9.79).

**Table 2.** Characteristics of Outpatients With Unspecified Community-Acquired Pneumonia Who Underwent a Fungal Diagnostic Test, by Coccidioidomycosis and Histoplasmosis Diagnosis Status

Characteristic	Coccidioidomycosis				Histoplasmosis			
	Patients, No. (%)		Bivariate *P* value	aOR (95% CI)	Patients, No. (%)		Bivariate *P* value	aOR (95% CI)
	Positive diagnosis (n = 616)	Negative diagnosis (n = 25 206)			Positive diagnosis (n = 94)	Negative diagnosis (n = 25 728)		
Demographic characteristics								
Age, median (IQR), y	50 (39-58)	55 (43-63)	<.001	NA	55 (40-62)	55 (43-63)	.25	NA
Age group, y								
18-44	236 (38)	6850 (27)	<.001	NA	29 (31)	7057 (27)	.66	NA
45-64	347 (56)	13 224 (52)			49 (52)	13 522 (53)		
≥65	33 (5)	5132 (20)			16 (17)	5149 (20)		
Sex								
Male	362 (59)	11 101 (44)	<.001	1 [Reference]	52 (55)	11 411 (44)	.03	1 [Reference]
Female	254 (41)	14 105 (56)		0.56 (0.47-0.67)	42 (45)	14 317 (56)		0.62 (0.41-0.93)
Census region (n = 24 747)								
Midwest	28 (5)	6748 (28)	<.001	1.18 (0.64-2.17)	55 (59)	6721 (27)	<.001	2.54 (1.64-3.95)
Northeast	5 (1)	3030 (13)		0.47 (0.18-1.22)	4 (4)	3031 (12)		0.40 (0.14-1.13)
South	31 (5)	10 256 (42)		1 [Reference]	32 (34)	10 255 (42)		1 [Reference]
West	550 (90)	4099 (17)		32.99 (22.7-47.95)	3 (3)	4646 (19)		0.21 (0.06-0.68)
Urban or rural classification (n = 20 640)								
Nonrural	593 (99)	16 503 (82)	<.001	1 [Reference]	67 (87)	17 029 (83)	.33	NA
Rural	5 (1)	3539 (18)		0.13 (0.05-0.32)	10 (13)	3534 (17)		
Index date season								
Spring	109 (18)	6439 (26)	<.001	1 [Reference]	13 (14)	6535 (25)	.001	1 [Reference]
Summer	168 (27)	6018 (24)		1.63 (1.25-2.13)	24 (26)	6162 (24)		1.90 (0.97-3.76)
Fall	226 (37)	6288 (25)		2.23 (1.73-2.88)	39 (41)	6475 (25)		2.73 (1.45-5.14)
Winter	113 (18)	6461 (26)		1.02 (0.76-1.36)	18 (19)	6556 (25)		1.36 (0.66-2.78)
Underlying conditions on or in the 90 d before index date								
Asthma	50 (8)	3786 (15)	<.001	0.54 (0.39-0.74)	10 (11)	3826 (15)	.25	NA
Autoimmune inflammatory disease	32 (5)	1610 (6)	.23	NA	16 (17)	1626 (6)	<.001	3.00 (1.72-5.21)
Chronic obstructive pulmonary disease or other chronic lower respiratory disease	35 (6)	5389 (21)	<.001	0.42 (0.29-0.61)	20 (21)	5404 (21)	.95	NA
Diabetes	60 (10)	4570 (18)	<.001	0.72 (0.57-0.91)	11 (12)	4619 (18)	.12	NA
Hyperlipidemia	70 (11)	4487 (18)	<.001	NA	16 (17)	4541 (18)	.87	NA
Hypertension	114 (19)	9371 (37)	<.001	NA	25 (27)	9460 (37)	.04	0.49 (0.31-0.79)
Hypothyroidism	34 (6)	2407 (10)	<.001	NA	8 (9)	2433 (9)	.75	NA
Immunosuppression	46 (7)	4394 (17)	<.001	0.49 (0.35-0.69)	22 (23)	4418 (17)	.02	NA
HIV	2 (<1)	201 (1)	.25	NA	3 (3)	200 (1)	.04	NA
Cancer	19 (3)	2709 (11)	<.001	NA	8 (9)	2720 (11)	.52	NA
Immunosuppressive medication besides prednisone^a^	17 (3)	930 (4)	.23		9 (10)	938 (4)	.008	NA
Prednisone (≥21 d supply)	12 (2)	1197 (5)	.001	NA	8 (9)	1201 (5)	.08	NA
Solid-organ or stem cell transplant	3 (<1)	527 (2)	.006	NA	2 (2)	525 (2)	.72	NA
Overweight and obesity	54 (9)	3417 (14)	.001	NA	8 (9)	3463 (13)	.16	NA
Pregnancy	1 (<1)	169 (1)	.20	NA	0	170 (1)	>.99	NA
Smoking (current or past)	40 (6)	4414 (18)	<.001	0.64 (0.45-0.90)	18 (19)	4436 (17)	.63	NA
Selected symptoms and clinical findings on or in the 90 d before index date								
Symptoms								
Abnormal weight loss	5 (1)	478 (2)	.05	NA	12 (13)	471 (2)	<.001	5.15 (2.71-9.79)
Chest pain	182 (30)	6108 (24)	.002	1.65 (1.35-2.02)	35 (37)	6255 (24)	.004	1.84 (1.20-2.82)
Chills, without fever	7 (1)	266 (1)	.85	NA	4 (4)	269 (1)	.02	3.99 (1.43-11.17)
Cough	313 (51)	12 131 (48)	.19	NA	52 (55)	12 392 (48)	.17	NA
Dyspnea	159 (26)	9171 (36)	<.001	NA	36 (38)	9294 (36)	.66	NA
Erythema nodosum	11 (2)	10 (<1)	<.001	NA	0	21 (<1)	>.99	NA
Fatigue or malaise	68 (11)	2826 (11)	.89	NA	17 (18)	2877 (11)	.03	NA
Fever	173 (28)	5290 (21)	<.001	1.30 (1.06-1.6)	22 (23)	5441 (21)	.59	NA
Generalized hyperhidrosis	218 (35)	10 (<1)	.047	NA	0	228 (1)	>.99	NA
Myalgia	38 (6)	905 (4)	.001	1.66 (1.11-2.49)	4 (4)	939 (4)	.78	NA
Pain in joint	54 (9)	3069 (12)	.01	NA	9 (10)	3114 (12)	.45	NA
Rash	55 (9)	545 (2)	<.001	3.24 (2.27-4.63)	0	600 (2)	.29	NA
Clinical findings								
Acute respiratory failure	13 (2)	1848 (7)	<.001	0.52 (0.28-0.97)	4 (4)	1857 (7)	.27	2.14 (1.41-3.25)
Diseases of mediastinum	1 (<1)	65 (<1)	>.99	NA	1 (1)	65 (<1)	.21	NA
Enlarged lymph nodes	35 (6)	1068 (4)	.08	1.74 (1.15-2.63)	10 (11)	1093 (4)	.007	NA
Eosinophilia	27 (4)	1643 (7)	.03	NA	6 (6)	1664 (6)	.97	NA
Hypoxemia	11 (2)	1966 (8)	<.001	0.44 (0.23-0.83)	8 (9)	1969 (8)	.76	NA
Other nonspecific abnormal finding of lung field	190 (31)	8103 (32)	.49	NA	50 (53)	8243 (32)	<.001	NA
Pleural effusion	47 (8)	2560 (10)	.04	NA	4 (4)	2603 (10)	.06	0.29 (0.11-0.81)
Solitary pulmonary nodule	23 (4)	1311 (5)	.10	NA	12 (13)	1322 (5)	.003	NA
No. of symptoms and clinical findings, median (IQR)	2 (1-3)	2 (1-3)	.60	NA	3 (2-4)	2 (1-3)	<.001	NA
Practitioner type on the index date^b^								
Family practitioner or internal medicine	235 (38)	10 252 (41)	.21	NA	36 (38)	10 451 (41)	.65	NA
Pulmonologist	17 (3)	1547 (6)	.001	NA	9 (10)	1555 (6)	.15	NA
Nurse practitioner or physician assistant	76 (12)	2456 (10)	.03	NA	6 (6)	2526 (10)	.26	NA
Acute care hospital	246 (40)	14 382 (57)	<.001	0.75 (0.62-0.92)	59 (63)	14 569 (57)	.23	NA
Infectious disease specialist	6 (1)	344 (1)	.41	NA	3 (3)	347 (1)	.14	NA
Emergency department or urgent care	245 (40)	8527 (34)	.002	NA	38 (40)	8734 (34)	.19	NA
None of the above practitioner types	65 (11)	2169 (9)	.09	0.63 (0.47-0.86)	3 (3)	2231 (9)	.06	NA
Received antibiotics from >1 class	336 (55)	10 923 (43)	<.001	1.40 (1.17-1.67)	39 (41)	11 200 (44)	.68	NA
Underwent chest imaging	477 (77)	19 747 (78)	.59	NA	78 (83)	20 146 (78)	.27	NA

Abbreviations: aOR, adjusted odds ratio; NA, not applicable.

^a^ Includes tumor necrosis factor–α inhibitors, interleukin-17 and interleukin-23 inhibitors, mycophenolate mofetil, and tacrolimus.

^b^ Categories are not mutually exclusive.

### Other Less-Common CAP Causes

Regardless of fungal diagnostic testing receipt, 1066 of the 573 994 patients with unspecified CAP received a diagnosis code for blastomycosis, coccidioidomycosis, or histoplasmosis within 90 days after the index date. Other selected diagnoses in the postindex period included pneumococcal pneumonia (1727 patients), *P aeruginosa* pneumonia (1234 patients), MRSA pneumonia (819 patients), and legionellosis (527 patients).

## Discussion

In this cohort study of commercial health insurance claims data, testing for blastomycosis, coccidioidomycosis, and histoplasmosis was uncommon (<5%) among adult outpatients with unspecified CAP. In contrast, nearly one-third of patients with unspecified CAP received antibiotics from multiple classes, possibly signifying a lack of symptom resolution after 1 antibiotic course and indicating that these patients likely fit the criteria for consideration of fungal disease testing,^1^ given that nearly every continental US state is endemic for at least 1 of these diseases. Among patients who underwent fungal disease testing, approximately 3% received a blastomycosis, coccidioidomycosis, or histoplasmosis diagnosis code. These fungal diseases may be more common causes of CAP than previously appreciated.^9^

The fungal disease testing rates we observed are comparable to those in a nationwide survey of primary care practitioners’ self-reported fungal disease testing practices for patients with CAP, in which 3.7% and 2.8% of practitioners reported frequently testing patients with CAP for coccidioidomycosis and histoplasmosis, respectively.^3^ This survey also revealed higher testing rates in regions and states with higher fungal disease burden, similar to our results showing increased testing in the West (likely for coccidioidomycosis) and the Midwest (likely for histoplasmosis); however, some *CPT* codes for fungal serologic tests are not specific enough to enable analyses of testing rates for each disease separately.

Our results showing that 17% of patients with CAP in Arizona and 6% in California underwent fungal testing were similar to those of previous retrospective studies showing coccidioidomycosis testing rates of 1% to 23% in Arizona and 6% in California.^6,15,16^ Among tested patients, the proportion who received a fungal diagnosis in our study (23% in Arizona and 10% in California) was also similar to those in previous studies, which showed 11% to 32% test positivity in Arizona and 8% to 18% in California.^6,15,16^ State-specific fungal disease testing and diagnosis patterns among patients with CAP outside of these highly coccidioidomycosis-endemic areas have not previously been described; our results can serve as a benchmark for future studies, particularly to more precisely evaluate the proportion of CAP caused by histoplasmosis and blastomycosis in the Midwest. A notable limitation of our analysis is that the insurance policy holder’s state might not reflect patients’ exposure location, and some diagnoses made outside the known geographic range for each disease likely represent travel-associated cases.^17,18^ This highlights the importance for clinicians to obtain patients’ travel history and testing for fungal diseases based on exposure to known endemic areas, as recommended in recent fungal disease-specific clinical guidance.^1^

Among patients with unspecified CAP, blastomycosis, coccidioidomycosis, and histoplasmosis combined were approximately twice as common as legionellosis in our analysis. This finding is also supported by national public health surveillance data, which show 17 612 coccidioidomycosis cases and 7512 legionellosis cases reported in 2022 (the most recent year of finalized surveillance data), despite coccidioidomycosis reporting being less complete than legionellosis reporting.^19^ Similarly, fungal diseases in our analysis were more common than MRSA pneumonia, which has been previously estimated to account for 2.4% of CAP cases among hospitalized adults.^20^ On the basis of these relative frequencies, incorporating recommendations for disease testing for blastomycosis, coccidioidomycosis, and histoplasmosis into national CAP diagnosis and treatment guidelines is critical to increase clinician awareness and detection of these diseases and knowledge of which patients with CAP would benefit from fungal disease testing.

Our results show that fungal disease testing was more commonly performed for patients with underlying conditions (eg, immunosuppression, autoimmune disease, or COPD) that are known risk factors for blastomycosis, coccidioidomycosis, and histoplasmosis or for severe forms of these diseases; this encouraging finding may indicate that health care practitioners are aware of the increased risk that these diseases pose for patients with certain underlying conditions. We also identified clinical factors associated with fungal disease testing, several of which (eg, rash or receipt of multiple antibiotics) have previously been associated with coccidioidomycosis testing among patients with CAP.^6,15^ Certain uncommon symptoms and clinical findings, such as erythema nodosum, lymphadenopathy, hyperhidrosis, and solitary pulmonary nodule, were also significantly associated with undergoing fungal disease testing and have previously been shown to be particularly indicative of fungal diseases.^16,21^ This could suggest that health care practitioners are aware that these signs and symptoms can be specific for fungal diseases and are testing accordingly, or alternatively, that fungal disease testing was more commonly performed for patients with these signs and symptoms simply because nonfungal CAP causes had already been ruled out. The latter explanation may be more likely, on the basis of the missed opportunities for fungal disease testing we observed (median of health care 3 visits between the CAP index date and testing) and the finding of higher odds of testing among patients who received antibiotics from multiple classes. Finally, patients’ likelihood of undergoing fungal disease testing may be further influenced by factors not captured by our study, such as health care practitioner experience and continuing medical education sources.^3^

Several factors (rash, myalgia, and multiple antibiotics) positively associated with receiving a coccidioidomycosis diagnosis code in our analysis support previous studies’ findings.^6,11,12^ Factors associated with histoplasmosis diagnosis among patients with CAP have not been previously studied, but in enhanced public health surveillance, autoimmune inflammatory disease was the most common underlying condition (possibly reflecting likely reactivation or acquisition in the setting of immunosuppressive therapy), and chest pain and weight loss were frequent symptoms.^22^

### Limitations

Our study’s primary limitations are the potential for misclassification inherent in medical coding data and the lack of laboratory result data. Incomplete capture of fungal disease testing represented by *CPT* codes may explain the discrepancy between the number of patients with CAP who both underwent fungal testing and received a fungal diagnosis code (755 patients) vs those who only received a fungal diagnosis code (1066 patients). Specifically, we might have underestimated the proportion of patients tested by culture because only fungal-specific culture *CPT* codes were included, but fungi can also grow on bacterial cultures. Other limitations not previously mentioned include the inability to calculate state-specific proportions of patients who received diagnoses with each fungal disease separately because of small sample sizes. Furthermore, the dataset does not contain data on race and ethnicity, which has been previously associated with variation in testing practices for coccidioidomycosis and test positivity among patients with CAP in Southern California.^6^

## Conclusions

In conclusion, this analysis of commercial insurance claims data provides baseline estimates of nationwide and state-specific testing rates for blastomycosis, coccidioidomycosis, and histoplasmosis among adult outpatients with CAP in the US. Inclusion of fungal-specific testing into national guidelines for diagnosis and treatment of CAP may help increase timely testing for fungal diseases among patients with CAP and improve patient outcomes. Increased recognition of these diseases would also serve to decrease inappropriate antibacterial use and potentially reduce health care utilization.

## Notes

Supplement 1. eTable. *International Classification of Diseases, Tenth Revision, Clinical Modification (ICD-10-CM)* and *Current Procedural Terminology (CPT)* Codes Used to Identify Conditions and Procedures of Interest

Supplement 2. Data Sharing Statement
