# Supplementary material for: Blastomycosis, Histoplasmosis, and Coccidioidomycosis in Outpatient Community-Acquired Pneumonia
Source: JAMA Netw Open. 2026 Jan 14;9(1):e2553965. doi: 10.1001/jamanetworkopen.2025.53965 (PMC12805450; doi:10.1001/jamanetworkopen.2025.53965)
Supplement: Supplement 1. — eTable. International Classification of Diseases, Tenth Revision, Clinical Modification (ICD-10-CM) and Current Procedural Terminology (CPT) Codes Used to Identify Conditions and Procedures of Interest [file jamanetwopen-e2553965-s001.pdf]

# Supplemental Online Content

Benedict K, Thompson GR III, Ampel NN, Smith DJ, Toda M, Hennessee I. Blastomycosis, histoplasmosis, and coccidioidomycosis in outpatient community-acquired pneumonia. *JAMA Netw Open*. 2026;9(1):e2553965. doi:10.1001/jamanetworkopen.2025.53965

**eTable.** *International Classification of Diseases, Tenth Revision, Clinical Modification (ICD-10-CM)* and *Current Procedural Terminology (CPT)* Codes Used to Identify Conditions and Procedures of Interest

This supplemental material has been provided by the authors to give readers additional information about their work.

© 2026 Benedict K et al. *JAMA Network Open*.

**eTable.** ***International Classification of Diseases, Tenth Revision, Clinical Modification (ICD-10-CM)* and *Current Procedural Terminology (CPT)* Codes Used to Identify Conditions and Procedures of Interest**

| **Description** | ***ICD-10-CM* code(s)** |
|---|---|
| Blastomycosis | B40 |
| Coccidioidomycosis | B38 |
| Histoplasmosis | B39 |
| Unspecified pneumonia | J12.89, J12.9, J15.8, J15.9, J16.8, J18.0, J18.1, J18.8, J18.9 |
| Legionnaires' disease | A48.1 |
| Pneumonia due to *Streptococcus pneumoniae* | J13 |
| Pneumonia due to *Pseudomonas* | B96.5, J15.1 |
| Pneumonia due to methicillin-resistant *Staphylococcus aureus* | A49.02, J15.212 |
| **Compatible symptoms** | |
| Abnormal weight loss | R63.4 |
| Chest pain | R07 |
| Chills, without fever | R68.83 |
| Cough | R05 |
| Dyspnea | R06 |
| Erythema nodosum | L52 |
| Fatigue or malaise | R53.8 |
| Fever | R50.81, R50.9 |
| Generalized hyperhidrosis | R61 |
| Myalgia | M79.1 |
| Pain in joint | M25.5 |
| Rash | R21 |
| **Compatible clinical findings** | |
| Acute respiratory failure | J96.0 |
| Diseases of mediastinum | J98.5 |
| Enlarged lymph nodes | R59 |
| Hypoxemia | R09.02 |
| Other nonspecific abnormal finding of lung field | R91.8 |
| Pleural effusion | J90 |
| Solitary pulmonary nodule | R91.1 |
| Eosinophilia | D72.1, D72.82 |
| **Underlying conditions** | |
| Asthma | J45 |
| Autoimmune inflammatory diseases (multiple sclerosis, myasthenia gravis, inflammatory bowel disease, psoriasis, lupus, rheumatoid arthritis, and ankylosing spondylitis) | G35, G70, K50, K51, L40, L93, M05, M06, M08, M33, M35.2, M45 |
| Cancer | C00–C96, excluding C44 |
| Chronic obstructive pulmonary disease (COPD) or other chronic lower respiratory disease | J40-47, excluding J45 |
| Diabetes | E08–E13 |
| HIV/AIDS | B20, Z21 |
| Hypertension | I10 |

© 2026 Benedict K et al. *JAMA Network Open*.

| | |
|---|---|
| Hyperlipidemia | E78.5 |
| Hypothyroidism | E03.8 |
| Overweight and obesity | E66 |
| Pregnancy | Z33.1, Z33.3, Z34, O09 |
| Solid organ or stem cell transplant | T86, Z94 (excluding Z94.7), Z95.2, Z95.3 |
| Smoking (current or past) | F17, Z87.891 |
| **Description** | ***CPT* codes** |
| Chest CT | 71250, 71260, 71270 |
| Chest x-ray | 71045, 71046, 71047, 71048 |
| Fungal culture | 87102, 87103, 87106, 87107 |
| Fungal microscopy | 87205, 87206, 87210 |
| Fungal serology | 86171, 86329, 86331, 86612, 86698, 87385, 87449, 86635 |

© 2026 Benedict K et al. *JAMA Network Open*.
