# Supplementary material for: Blastomycosis, Histoplasmosis, and Coccidioidomycosis in Outpatient Community-Acquired Pneumonia
Source: JAMA Netw Open. 2026 Jan 14;9(1):e2553965. doi: 10.1001/jamanetworkopen.2025.53965 (PMC12805450; doi:10.1001/jamanetworkopen.2025.53965)
Supplement: Supplement 2. — Data Sharing Statement [file jamanetwopen-e2553965-s002.pdf]

Data Sharing Statement

Benedict. Blastomycosis, Histoplasmosis, and Coccidioidomycosis in Outpatient Community-Acquired Pneumonia. *JAMA Netw Open*. Published January 14, 2026.
doi:10.1001/jamanetworkopen.2025.53965

Data
Data available: No

Additional Information
Explanation for why data not available: This study used third-party data that we cannot legally distribute. All relevant summary data are within the manuscript and the supporting files. The raw data underlying the results presented are available from the MerativeTM MarketScan® Research Databases: https://www.merative.com/documents/brief/marketscan-explainer-general. Others can access the data by going to this website and contacting Merative.
